# Extreme Minuteness

**Published:** 1884-11

**Authors:** 


### EXTREME MINUTENESS.

WHEN vision is not aided by any magnifying process, there is a point of minuteness, as all know, when an object will make no impression upon the retina, and will not be seen by the unaided eye. But when the object is viewed by means of a microscope, it becomes visible. There is a question, however, that remains unanswered, which is, whether any object may become so attenuated that it cannot be made visible by any means. Not many years ago, less probably than twenty-five, there were lines that could not be resolved by any microscope lenses then in existence, which can be exhibited now without any difficulty; but at the time, makers of lenses had not attained to the skill of making them with large angles of aperture, but now they are made with the highest angle that is possible, and consequently the capacity of such objectives can only be increased by greater skill in their manufacture. But the limit of angle of aperture having been reached—no opportunity remaining of increasing capacity in that direction—is it not reasonable to suppose that, with present appliances, no greater skill in manufacture can be expected? Sir Royston Pigott, recently, at a meeting of the R. M. S., stated that he had seen globules of mercury, made by smashing a minute particle of mercury with a watch spring, less than the millionth of an inch. Another member replied that he was not aware that there is any limit of visibility in the microscope other than that imposed by the sensibility of the observer's retina, the correction of the objective, and the illumination.—*The Microscope.*

